# Retrobulbar metastasis and intracranial invasion from postoperative hepatocellular carcinoma: A case report and review of the literature

**DOI:** 10.3892/ol.2014.2733

**Published:** 2014-11-24

**Authors:** CHUN-YONG CHEN, JIAN-HONG ZHONG, JING-LI LIU

**Affiliations:** 1 Department of Neurology, The First Affiliated Hospital, Guangxi Medical University, Nanning, Guangxi 530021, P.R. China; 2 Department of Hepatobiliary Surgery, The Affiliated Tumor Hospital, Guangxi Medical University, Nanning, Guangxi 530021, P.R. China

**Keywords:** hepatocellular carcinoma, retrobulbar metastasis, intracranial invasion, meningioma

## Abstract

Hepatocellular carcinoma (HCC) is the most common malignant cancer of the liver and the third ranking cause of cancer-related mortality worldwide. Following the diagnosis of HCC, intrahepatic and extrahepatic metastasis patients account for ~50–75% of all HCC cases, lung and regional lymph nodes metastasis are the most common; metastasis to bone, skin and adrenal glands are rare, orbit metastasis and intracranial invasion are extremely rare. The present study reports the rare case of a patient with HCC that metastasized to the head. The patient presented with retrobulbar and intracranial invasion, and sub-scalp extension. Based on imaging findings, the lesion was initially misdiagnosed as meningioma, however, postoperative pathological examinations resulted in a definitive diagnosis of HCC metastasis. Based on the present case and a review of the relevant literature published since 2009, the current study recommends that metastasis must be considered when diagnosing retrobulbar head lesions in patients with HCC, regardless of contradictory imaging findings and other clinical indicators, which may closely mimic the original head lesion.

## Introduction

Hepatocellular carcinoma (HCC) is the sixth most common type of cancer and third most common cause of cancer-related mortality worldwide. A total of ~50,000 new cases are estimated to occur annually (1). With an advanced diagnosis and effective early treatment via curative resection, ablation or transplantation, the median survival time of patients is >5 years (2). HCC is strongly associated with chronic hepatitis B virus (HBV) infection; just over half of HCC patients worldwide are HBV-infected and the proportion is much higher in Asia (1). A total of 80% of HCC patients from Asia and South Africa present with risk factors including HBV and aflatoxin B1 exposure (2). Furthermore, a substantial proportion of HCC patients (range, 14–37%) develop extrahepatic HCC metastasis (3). Such metastasis most commonly occurs in adjacent lymph nodes and the lungs, and less frequently in the bones, adrenal glands and brain (4). Surgical resection is a safe and effective strategy for the treatment of numerous patients with primary or metastatic HCC, however, recurrence is common, with ≤70% of patients exhibiting tumor recurrence within five years of curative surgery (5). The present study describes an unusual case of a patient with chronic HBV infection and HCC who, five months following surgical resection, presented with a retrobulbar and intracranial lesion. Although the lesion mimicked meningioma, subsequent pathological analysis identified it as HCC metastasis. Additionally, the patient did not show any evidence of tumor recurrence in the liver. Written informed consent for the publication of this study was obtained from the patient.

## Case report

A 43 year-old male was admitted to the Neurosurgery Department of the First Affiliated Hospital of Guangxi Medical University (Nanning, China) on the 8 April, 2014 with approximately a one-month history of mild headache and mild protopsis of the left eye. Upon physical examination, the patient exhibited mild protopsis of the left eye and ipsilateral visual acuity of 0.3 (normal range, 1.0–2.0) and contralateral visual acuity of 1.5 (normal range, 1.0–2.0). A frontal mass under the scalp adjacent to the left orbit was visible, however, no pulsation or other positive clinical manifestations were observed on palpation. Furthermore, the patient reported no abnormal sensations around the left eyeball, vomiting or vertigo.

**Figure 1:**
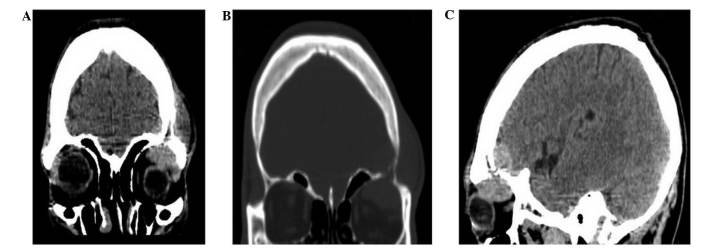
Computed tomography imaging of the patient, including: (A) A coronal plane image demonstrating intracranial and extracranial masses in the left orbit region under the scalp; (B) a coronal plane image indicating lytic changes in the skull bone; and (C) a sagittal plane image showing a cranio-orbital mass.

**Figure 2:**
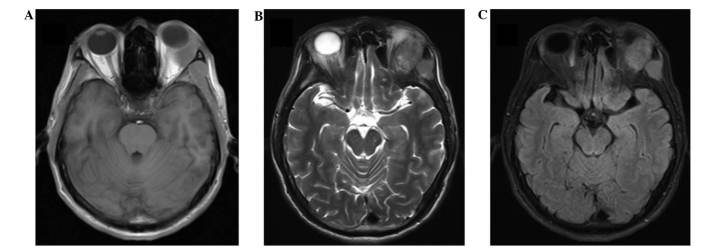
Magnetic resonance imaging of the patient. The isointensity of the lesion is visible on (A) T1-weighted, (B) T2-weighted and (C) fluid-attenuated inversion recovery images.

**Figure 3:**
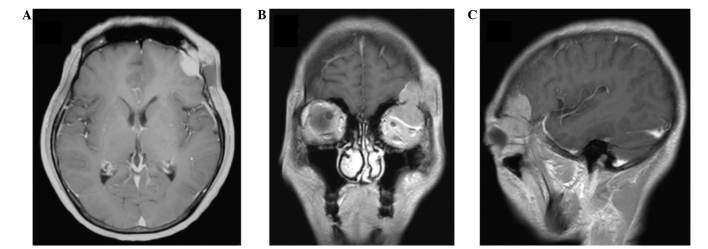
T1-enhanced magnetic resonance imaging of the patient revealed (A) dural attachment and a mild dural tail sign, and (B) coronal and (C) saggital plane images revealed a cranio-orbital invasive lesion with moderate enhancement.

Computed tomography (CT) and cerebral magnetic resonance imaging (MRI) scans identified retrobulbar metastasis and intracranial invasion with lytic changes in the left orbital and temporal bones (Fig. 1). Almost homogeneous isointensity was visible on T1- and T2-weighted images, as well as on fluid-attenuated inversion recovery images (Fig. 2). Additionally, a dural tail sign with lytic changes in the adjacent skull bones was present on T1-enhanced MRI images (Fig. 3). Therefore, a diagnosis of meningioma with retrobulbar and under-scalp invasion was determined.

**Figure 4:**
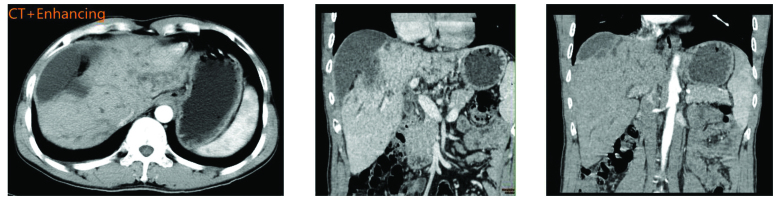
Multiple plane enhanced computed tomography of the upper abdomen revealed no recurrent hepatocellular carcinoma lesion in the liver.

An enhanced CT scan of the upper abdomen revealed no recurrent hepatic lesions (Fig. 4), however, serological analysis demonstrated that the patient was positive for hepatitis B (HB) surface antibody (Ab), HB e Ab and HB core Ab. Additionally, the patient exhibited an α-fetoprotein level of 29.07 ng/ml (normal range, 0–11 ng/ml), however, no abnormalities were detected in the liver biochemistry or in other routine tests.

**Figure 5:**
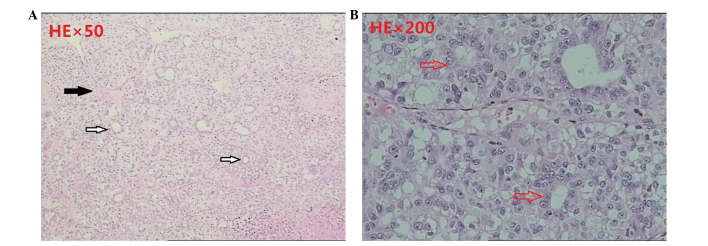
Postoperative pathology of the excised metastatic lesion, showing (A) the portal area (black arrow), bile ducts (white arrow; magnification, ×50); and (B) nest-shaped cell clusters with central lumen that adopted the shape of the bile ducts (arrow; magnification, ×200; hematoxylin and eosin staining).

Due to these findings, the intracranial mass was surgically resected. Following scalp incision, a round mass with the appearance of fish meat and a diameter of 5 cm was identified to have invaded the local skull area. It was tightly adhered to the dura mater and protruded into the left orbit. Pathological examination of the lesion following hematoxylin and eosin staining was used to identify the mass as HCC (Fig. 5). Therefore, the patient was definitively diagnosed with retrobulbar metastasis and intracranial invasion from postoperative hepatocellular carcinoma (HCC).

## Discussion

The combination of MRI imaging results and the absence of detectable HCC lesions on the liver by enhanced CT of the upper abdomen resulted in an initial misdiagnosis of meningioma with retrobulbar invasion. Meningiomas are the second most common type of primary brain tumor, accounting for 15–20% of primary brain tumors; the intracranial mass protrudes extracranially via the bones and mimics thickened bone (6). Ultimately, surgical findings and postoperative pathology revealed the mass in the present patient to be a HCC metastatic lesion.

The current study conducted a review of the relevant Chinese- and English-language medical literature, and identified 39 cases of HCC metastasis to the head reported since 2009 (Table I) (4,7–35). Of these 39 patients, 11 exhibited peri-orbital metastasis similar to that of the present patient (Table II) (4,17–19,25,30). Of the 11 cases with HCC metastasis to the orbit published since 2009, all were male (with the exception of no gender being reported in one case)e, five exhibited invasion from the right side, only two patients demonstrated peri-orbital invasion combined with cerebral invasion, yet cerebral invasion also occurred in the present case. Painful proptosis was commo; seven patients suffered from this, as well as the patient in the present case. Two patients had not been diagnosed with HCC with metastasis when they were hospitalized. The metastatic mass invaded the cerebrum in only two cases and two patients experienced metastasis to the head five years following liver transplantation to treat HCC (28,33). The first case was a 70-year-old male, who had HCC and underwent liver transplantation in August 2006, and was without HCC recurrence in the following 4 years. However, the patient was identified to have a 3-cm painless, subcutaneous mass; CT scans showed an osteolytic lesion in the frontal bone without dural matter invasion, which was pathologically diagnosed as HCC metastasis. The second case was a 38-year-old male, who underwent surgery to remove a liver mass and received a liver transplantation 4 months previously; however, a well-defined localized painless mass was identified on the right temporal side of the patient’s scalp. MRI showed that the mass involved the outer and inner skull tables, and was attached to the dura matter. Pathological analysis led to a diagnosis of HCC metastasis.

The present study illustrates that extrahepatic metastasis can occur with or without concurrent intrahepatic lesions following curative resection for HCC. Surgery is widely considered to be an effective treatment strategy for extrahepatic metastatic lesions from HCC; however, systemic sorafenib therapy may be another possible treatment modality, as it has demonstrated improved survival in HCC metastatic patients (36).

In conclusion, the present study highlights the importance of considering metastasis in the differential diagnosis of patients with a history of HCC who present with an intracranial mass. The possibility of HCC metastasis must be acknowledged despite the results of imaging procedures and other clinical indicators, as brain metastases may closely mimic meningioma.

## Figures and Tables

**Table I:** Location of hepatocellular carcinoma metastases to the head reported in the Chinese- and English-language literature since 2009.

Lesion location	n (%)
Orbit	11 (28.2)
Face	8 (20.5)
Skull base	5 (12.8)
Scalp	4 (10.3)
Meninges	4 (10.3)
Calvaria	3 (7.7)
Cerebrum	2 (5.1)
Dura and scalp	2 (5.1)
Total	39 (100)

**Table II:** Clinical characteristics of patients with HCC metastasis to the orbit published since 2009.

Study Author, year	Gender/Age, years	Side	Cerebrum invasion	Other metastasis	Clinical presentation	Risk factor for metastasis
Quick *et al*, 2009	M/52.0	R	No	No	Proptosis and diplopia	No
Kolarevic *et al*, 2011	M/70.0	R	No	Spleen, maxillary, retroperitoneal lymph node	Bleeding difficult to control after dental surgery	No
Mustapha *et al*, 2011	M/25.0	R	No	No	Progressive pain, swelling and blurred vision for 1 month	NR
Guerriero *et al*, 2011	M/45.0	L	Yes	Lung	Proptosis for 1 month	HCC liver transplant 5 years previously
Piccirillo *et al*, 2013	NA	L	No	Lung	NR	HCC liver transplant 3 years previously
Eldesouky *et al*, 2013	M/62.0	L	No	No	Painful proptosis for 8 weeks	HCC for 1.5 years
	M/70.0	L	No	No	Painful proptosis for 6 weeks	HCV for 10 years
	M/55.0	R	No	No	Proptosis for 4 weeks	HCC for 1 year
	M/65.0	L	No	No	Painful proptosis for 2 weeks	HCV for 12 years
	M/47.0	L	No	No	Painful proptosis for 6 weeks	HCV for 15 years
	M/62.0	R	Yes	No	Eyelid ptosis for 8 weeks	HCC for 14 months
Present study	M/43.5	L	Yes	No	Mild headache and mild protopsis for 4 weeks	HCC for 5 months

M, male; R, right; L, left; HCC, hepatocellular carcinoma; HCV, hepatitis C virus infection; NR, not reported.
